# Recent advances and novel agents for gastrointestinal stromal tumor (GIST)

**DOI:** 10.1186/1756-8722-5-21

**Published:** 2012-05-08

**Authors:** Gurpreet Lamba, Samir Ambrale, Byung Lee, Ridhi Gupta, Shamudheen M Rafiyath, Delong Liu

**Affiliations:** 1 Division of Oncology/Hematology, New York Medical College and Westchester Medical Center, Valhalla, NY, 10595, USA

## Abstract

The discovery of CD117 mutation in almost all gastrointestinal stromal tumors (GISTs) marked a milestone. Other spindle cell neoplasms arising from the GI tract including lipoma, schwannoma, hemangioma, leiomyoma, and leiomyosarcoma are typically CD117-negative. GIST research and clinical care now represent a paradigm of translating discoveries in the molecular pathogenesis of cancer into highly effective targeted therapies that selectively inhibit etiologic “driver” pathways, leading to dramatically improved clinical outcomes. A series of investigations and trials are underway to develop novel and effective ways to treat patients with GIST. In this review, we discuss the highlights of recent advances and novel agents for GIST therapy.

## Introduction

**Figure 1:**
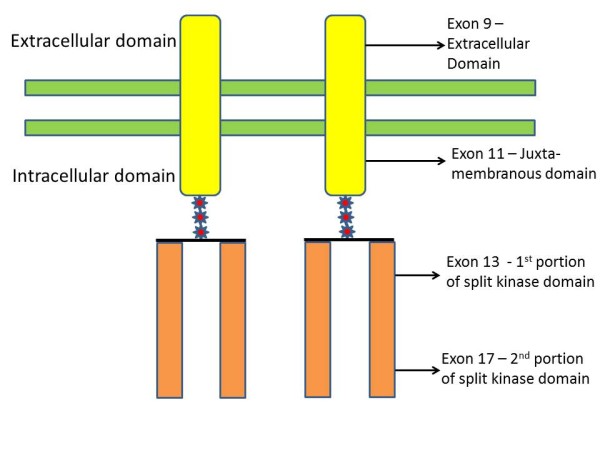
**KIT (CD117) receptor tyrosine kinase structure and common mutations found in gastrointestinal stromal tumor.** Arrows indicate the corresponding mutations in the exons.

Remarkable developments have occurred in gastrointestinal stromal tumor (GIST) research and clinical care in the past several years. GIST has served as a model for translational therapeutics in solid tumors. A major breakthrough occurred with the discovery of expression of the CD117 antigen by almost all GISTs. Other spindle cell neoplasms arising from the gastrointestinal (GI) tract including lipoma, schwannoma, hemangioma, leiomyoma, and leiomyosarcoma, are typically CD117-negative [1]. The CD117 molecule is part of the KIT (c-kit) receptor tyrosine kinase (KIT RTK) encoded by the KIT proto-oncogene (Figure 1). Since CD117 was found to be associated with GIST, the estimated incidence of GIST has been revised upward to approximately 5,000 new cases per year in the United States (US) [2,3].

## Molecular signature of GIST

**Figure 2:**
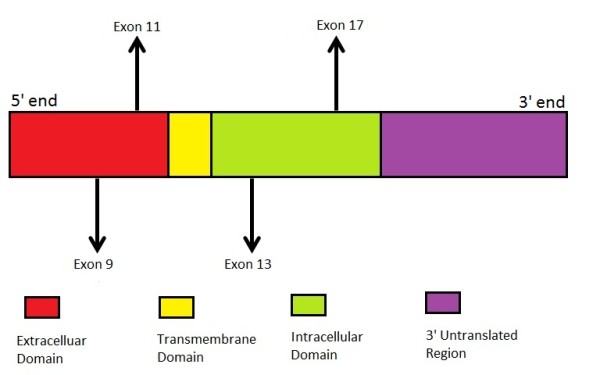
**KIT (CD117) gene structure and common mutations in gastrointestinal stromal tumor.** Arrows indicate the positions of common mutations in the KIT gene.

In 1998, Hirota *et al.* defined the relationship between GIST and certain mutations in the KIT proto-oncogene that conferred uncontrolled activation to the KIT signaling enzyme [4]. Importantly, almost all GIST lesions with mutant KIT demonstrate only a single site of mutation in the KIT gene (Figure 2). Complex genetic changes are rare at initial diagnosis. Gain-of-function mutations have been recognized most commonly (up to 70% of cases) in exon 11 of KIT. Approximately 15% of GIST patients do not demonstrate activation and aberrant signaling of the KIT receptor. An additional 10% harbor mutations in the platelet-derived growth factor receptor – alpha (PDGFRA) [5,6]. Very rare cases may have mutations in the BRAF kinase [7,8]. Overall, about 5% of GISTs have no detectable kinase mutations (and are often referred to as wild type GIST). Janeway and colleagues have also shown that germline mutation in succinate dehydrogenase subunits B, C or D can cause KIT-/PDGFRA- wild type GIST [9].

National Comprehensive Cancer Network (NCCN) guidelines recommend KIT immunostaining for all cases of suspected GIST, and if negative, mutational analysis [10,11]. Routine genotyping of KIT-positive GISTs is not recommended.

## Imatinib for metastatic, unresectable or recurrent GIST

Imatinib was found to be able to potently inhibit the tyrosine kinase activity of KIT. The United States (US)–Finland trial enrolled 147 patients with metastatic GIST between July 2000 and April 2001 [12]. Nearly concurrently, a dose-finding study was also begun in Europe under the auspices of the European Organization for Research and Treatment of Cancer (EORTC) Sarcoma Group to assess the tolerability and potential activity [13]. The two studies confirmed the unparalleled activity of imatinib in controlling metastatic GIST. The median overall survival (OS) of advanced GIST patients increased from 18 to 57 months with imatinib therapy [14]. Despite these excellent results complete responses (CR) are rare (less than 10 percent), and most patients who initially respond ultimately acquire resistance via additional mutations in KIT. The median time to progression is roughly two to three years [12,15-17], although it is longer in some series [18]. Factors influencing the duration of disease control are still not well understood [17].

Correlative studies have reported differences in the activity of imatinib based on the genotype of the GIST lesion. The mutations in KIT and PDGFRA correlate with clinical response [19-22]. In a report of 127 patients with GISTs receiving imatinib, activating mutations in KIT and PDGFRA were found in 88 and 4.7 per cent, respectively [19]. All of the KIT mutant isoforms were associated with a response, however only a subset of PDGFRA mutants were imatinib-sensitive. Among patients with KIT mutations, those with an exon 11 mutation had a significantly greater response rate compared to patients with an exon 9 mutation or no detectable mutation in KIT or PDGFRA (84 versus 48 and 0 per cent, respectively). Exon 11 mutation patients also exhibited a longer time to treatment failure. A US Intergroup trial subsequently confirmed these results. This trial enrolled 324 patients and compared the two doses of imatinib [22]. Patients whose tumors who had an exon 11 mutant isoform were more likely to have an objective response to imatinib than those with an exon 9 isoform or those who had no mutations (72 versus 44 and 45 per cent, respectively). Patients with an exon 11 mutation also had a significantly longer time to disease progression (25 versus 17 and 13 months, respectively) and OS (median 60 versus 38 and 49 months, respectively). This also translated into more durable disease control over time with continuous dosing of imatinib. The results of this trial as well as subset analysis from the randomized EORTC dose–response trial suggest that high dose imatinib may preferentially benefit patients with an exon 9 mutation [22,23]. In the EORTC trial, GISTs of 58 patients expressed an exon 9 mutant KIT protein. An initial daily imatinib dose of 800 mg resulted in a significantly superior progression-free survival (hazard ratio for progression 0.39) compared to 400 mg/day. In contrast, the time to progression was not affected by the initial dose in patients with an exon 11 mutation or wild-type KIT. No corresponding differences in overall survival between low-dose and high-dose initial therapy in patients with exon 9 mutations was seen. Similar conclusions were also reached in a meta-analysis that included patients treated on the EORTC and the US Intergroup trial [24]. Imatinib-sensitive PDGFRA mutations explain responses in certain GIST patients with wild-type KIT [19]. Thus, GIST lesion genotype is an important predictive tool and correlates with clinical efficacy of imatinib as a first-line therapy. In one large series of 289 GISTs with PDGFRA mutations, 181 (63 percent) had the imatinib-resistant substitution D842V. Because of the variability in response, patients with advanced GISTs should not be denied a trial of imatinib if they are KIT-negative. NCCN guidelines recommend initiating therapy for unresectable or metastatic disease with imatinib 400 mg daily [11]. However, if molecular diagnosis is available and the patient is exon 9-positive, they support the use of imatinib at 800 mg daily. In contrast, European Society for Medical Oncology recommends mutation testing for all patients and starting imatinib at 800 mg daily for exon 9 mutants [25].

Tyrosine kinase inhibitor (TKI) therapy does not appear to cure patients with metastatic GIST. Rapid disease progression was seen within months after the imatinib is stopped [26,27], this is considered a lifelong therapy. A French trial randomly assigned patients with advanced GIST and no disease progression after one year of imatinib to continuous treatment or interruption until disease progression [26]. The study was stopped prematurely after only 58 patients had been randomized when it became clear that the risk of progression was significantly higher if therapy was interrupted, even in completely responding patients.

At the 2011 annual meeting of American Society of Clinical Oncology (ASCO), A Le Cesne *et* reported the effect of interruption of imatinib therapy in patients with GIST enrolled on the BFR 14 trial [28]. GIST patients were randomly assigned to either interrupt or continue therapy with imatinib after 1, 3, and 5 yrs. Progression free survival was significantly lower in the patients that interrupted therapy as compared to the patients who continued therapy. Imatinib re-introduction allowed tumor control in 94% patients with interrupted treatment. There was no significant difference in time to secondary resistance or OS between both arms. At the same meeting, Domont *et al.* reported the influence of imatinib interruption and re-introduction on tumor burden in patients with GIST on the BFR 14 trial [29]. They found that imatinib interruption in responding patients with advanced GIST results in tumor progression even in patients who were in complete remission at randomization. Among patients with imatinib interruption 49% experienced progressive disease while 51% had new lesions with concomitant progression of known lesions. Thus, continuous therapy until disease progression (or lifelong if disease does not progress) is currently standard of care. These clinical data support the hypothesis that continuous and chronic exposure to imatinib is necessary to maintain control over a population of GIST cells that may remain quiescent in the long term as long as aberrant KIT signaling is inhibited. Future studies are required to assess whether periodic pulse therapy might suppress emergence of multidrug-resistant GIST clones.

## TKIs for imatinib-resistant GIST

Primary resistance was seen in 12 percent of 934 patients in the randomized European trial exploring two different doses of imatinib and was more likely in patients with lung but not liver metastases (41 percent) [16]. Alternatively, clonal evolution of resistant GIST may be detected after a durable objective response and disease control. Several mechanisms of resistance to imatinib in GIST have been explained [30,31]. Pharmacokinetic variability may also contribute to acquired drug resistance [32]. Limited clonal progression appears as the first sign of resistance to imatinib [31,33,34]. The mechanism of resistance to imatinib most commonly observed is the emergence of new secondary mutations [30,31]. Another likely mechanism is that pre-existing double-mutant tumor cells slowly grow out under the influence of chronic imatinib selection pressure, similar to the antibiotic-resistant strains of bacterial pathogens. Dose escalation of imatinib can also be considered in resistant patients started on imatinib 400 mg daily. The efficacy of this approach was shown in follow-up reports from both the American and European randomized dose-finding studies [14,35].

Sunitinib is an anti-angiogenesis agent by virtue of targeting multiple tyrosine kinases, including the vascular endothelial growth factor receptors (VEGFR) in addition to PDGFR [22,36-38]. An international phase III trial of sunitinib versus placebo in 312 patients with refractory disease definitively established the role of sunitinib in this setting [38] (Table 1). Patients demonstrating progression while on placebo crossed over to the active treatment arm. Despite a low objective response rate in the sunitinib group (7 percent partial response), median time to tumor progression, the primary endpoint, was fourfold longer as compared to the placebo group (27 versus 6 weeks). Despite the crossover, survival was also significantly better with initial sunitinib. Based on these data, this agent was approved for treatment of GIST following failure of imatinib in January 2006.

**Table 1:** TKIs for imatinib-resistant GIST

Study Drug	Disease	Clinical Trial	Number of patients	Results	Status	Reference
Sunitinib	Imatinib-resistant	Phase 3	312	TTP 27 weeks	FDA Approved. On NCCN guidelines.	[38]
Sorafenib	Imatinib- and Sunitinib resistant	Phase 2	38	ORR 68%	On NCCN guidelines.	[39]
Nilotinib	Imatinib- and Sunitinib resistant	Phase 3 ( ENEST g3)	248	No difference in PFS or OS	Further trials	[40]
Sorafenib	Imatinib- and Sunitinib resistant	Phase 2	41	ORR 37.6%	On NCCN guidelines.	[41]
Dasatinib	Imatinib- and Sunitinib resistant	Phase 1	47	PR - 32%	Further trials	[42]

Abbreviations: *TTP* time to progression; *ORR* overall response rate; *PR* partial response.

Clinical benefit (partial response or stable disease for longer than six months) was significantly higher for those with a primary KIT exon 9 (58 percent) or wild-type KIT/PDGFRA mutation (56 percent) than for those with a KIT exon 11 mutation (34 percent). The same pattern was seen for progression-free survival (PFS) and OS. Following progression on imatinib, patients with KIT exon 9 mutation or a PDGFRA mutation had a median time to progression of 19 months, while for those with exon 11 mutations, it was only 5 months. There was also a correlation between secondary mutations and response to sunitinib. Both progression-free and overall survival were significantly longer for patients with secondary KIT exon 13 or 14 mutations than for those with exon 17 or 18 mutations (7.8 versus 2.3 months). Resistance to sunitinib shares similar pathogenetic mechanisms to those identified in imatinib failure, with acquisition of secondary mutations after an extended initial response [43].

Limited data are available on the efficacy of sorafenib and other TKIs (i.e., dasatinib, motesanib, nilotinib) in refractory GIST or after resistance to imatinib and/or sunitinib [44-49]. The efficacy of sorafenib was addressed in a multicenter phase II trial involving patients with refractory GIST [50]. In a report presented at the 2011 ASCO GI Cancers symposium, the disease control rate was 68 percent, and median PFS was 5.2 months. The most common grade 3 toxicities were hand-foot syndrome and hypertension. Kindler and co-workers reported the final results at the 2011 ASCO Annual Meeting [39]. Thirty eight patients were enrolled with baseline mutations in exon 11 (65%), exon 9 (15%), PDGFRA (4%). They reported partial responses in 13% and stable disease in 55%. The median PFS was 5.2 months and OS was 11.6 months. Grade 3 and 4 toxicities included hand-foot syndrome (45%), hypertension (21%), diarrhea (8%), hypophosphatemia (8%), GI bleed (5%), thrombosis (3%), GI perforation (3%) and intracranial hemorrhage (3%).

Korean GIST Study Group (KGSG) reported the results of a prospective, multicenter, phase II study evaluating the efficacy and safety of sorafenib in patients with advanced GISTs who failed previous standard TKI’s [41]. Thirty-one patients with pathologically proven metastatic or unresectable GISTs who failed both imatinib and sunitinib were accrued. Ten patients received nilotinib as a third line treatment. With sorafenib, 3 patients (10%) achieved a partial response and 17 patients (55%) had stable disease. The median PFS was 4.9 months and the disease control rate was 37.6% at 6 months. Patients with prior use of 3rd line nilotinib and primary genotypes other than mutations at KIT exon 11 showed significantly worse PFS.

Guidelines from the NCCN suggest sorafenib as an option for patients with imatinib and sunitinib-resistant GIST [11]. Emerging results from *in vitro* studies suggest that the choice of salvage therapy in imatinib-refractory GISTs might depend, at least in part, on the specific mutation responsible for the acquisition of resistance [51]. However, these data require validation before they can be applied to clinical practice.

Nilotinib was studied in a randomized phase 3 clinical trial (ENEST g3) [40]. In this trial nilotinib was compared to a heterogeneous control arm in patients advanced/metastatic GIST who had failed imatinib and sunitinib. The control arm included best supportive care with physician choice to continue or stop imatinib or sunitinib. It failed to show significant benefit for nilotinib.

Dasatinib is an oral tyrosine kinase inhibitor of KIT, PDGFR, ABL and SRC with a distinct binding affinity for KIT and PDGFR. Trent and associates reported a phase II trial to assess antitumor activity of dasatinib in patients with advanced GIST who were refractory to imatinib and sunitinib [42]. They reported a partial response (PR) rate of 32% (15/47) by Choi criteria and 21% patients (10/47) were progression-free after 6 months. Median PFS and OS were 2.0 months and 19 months with median PFS for wild type GIST patients of 8.4 months. Dasatinib has significant activity but did not meet the predefined 6 month PFS rate of 30%.

## Recent advances and meeting updates

Several clinical trials are already in progress using next-generation agents that target the KIT receptor via different mechanisms or that target the alternate pathways. We will now review the highlights on GIST from the 2011 American Society of Clinical Oncology (ASCO) meeting and the 2011 ASCO – Gastro-intestinal cancers symposium (Table 2).

**Table 2:** New TKIs for GIST

Study drug	Disease	Dosage	Clinical Trial	Number of patients	Results	Reference
Regorafenib	Imatinib and Sunitinib Resistant	160 mg/day orally day 1–21 of 28 day cycle.	Phase 2	33	SD 86%	[52]
Masitinib	First line therapy	7.5 mg/kg/day	Phase 2	30	PFS 41 m	[53]
Crenolanib	Selective for D842V mutation	-	*In vitro*	-	Blocks the kinase activity of PDGFRA D842V mutants.	[54]
PTK787/ZK222584	Imatinib resistant	1,250 mg o.d	Phase 2	15	ORR 67%	[44]
AMG 706	Imatinib-resistant	600 mg daily	Phase 2	138	ORR33%	[48]

Abbreviations:*SD* stable disease; *PFS* progression free survival; *ORR* overall response rate.

## New TKIs

Regorafenib is a novel oral multi-kinase inhibitor which has a broad spectrum of antitumor activity in preclinical and early phase trials. George *et al.* conducted a multi-center phase II trial of regorafenib in patients with advanced GIST after prior therapy with at least imatinib and sunitinib [52]. Thirty three patients received at least one dose of study drug. Most common grade 3 treatment related toxicities were hypertension, hand-foot skin reaction, and hypophosphatemia. There were two grade 4 events, one hyperuricemia and one thrombosis. Most eligible patients were without disease progression after 4 cycles of regorafenib. Benefit was seen in patients whose tumors had primary KIT exon 11 mutations, KIT exon 9 mutations or wild type kinase genotype. Thus, regorafenib demonstrated significant activity in patients with advanced GIST previously treated with imatinib and sunitinib. An international phase III trial is currently underway in patients with advanced GIST following treatment with at least imatinib and sunitinib.

Masitinib is a new tyrosine kinase inhibitor which has a greater activity and selectivity than imatinib. It is an oral inhibitor of both the KIT and PDGFRA receptors. It may have greater activity than imatinib against wild-type GIST and juxta-membrane KIT mutants. Blay *et al.* evaluated the safety and efficacy of masitinib as a first line therapy in patients with imatinib-naïve, inoperable, locally advanced or metastatic GIST [53,55]. They reported a PFS of 41 months. OS was 72% at the end of 4 years. Main toxicities were rash (10%), neutropenia (7%) and abdominal pain (7%). A phase 3 trial is currently underway and actively recruiting participants (ClinicalTrials.gov identifier: NCT00812240).

Crenolanib (formerly CP-868596) is an orally bioavailable, highly potent and selective PDGFR TKI for the D842V mutation encoded by exon 18. Currently approved TKIs have little to no *in vitro* activity against this mutation and are thus clinically ineffective. Phase I trials of Crenolanib have shown a favorable toxicity profile, and achievable serum concentrations as high as 2,000 nanomolar [56]. At the recommended phase II dosage (ie, 100 mg twice daily with food), the steady-state serum concentrations were more than 16 nanograms/milliliter. The half life was in the range of 12.3 to 18.5 hours. Heinrich and associates reported on the effect of crenolanib on phosphorylation of the imatinib-resistant D842V PDGFRA activating mutation [54]. Mutant PDGFRα isoforms were expressed by transient transfection of Chinese hamster ovary cells and these transfected cells were treated with various concentrations of crenolanib or imatinib. Crenolanib was effective in blocking the activity of single or compound PDGFRA D842V mutant kinases. In contrast, imatinib had no significant activity against these same mutant kinases. A phase II clinical study of crenolanib for treatment of GIST patients with primary or secondary PDGFRA D842V mutation is currently recruiting patients (ClinicalTrials.gov Identifier: NCT01243346).

Motesanib (AMG706) is an oral inhibitor of VEGF, PDGF, and Kit receptors. In a phase 2 multicenter study of AMG 706 in 102 advanced imatinib-resistant GISTs [48], the objective response rate was 3%. This included 59% patients who had stable disease. PET scans showed an objective response rate of 30% and per Choi criteria of 41%. The median PFS was 16 weeks. The most common motesanib treatment-related grade 3 adverse events were hypertension (23%), fatigue (9%), and diarrhea (5%).

PTK787/ZK222584 is another novel, oral selective inhibitor of receptor tyrosine kinases (KIT, PDGFRs, VEGFR-1 and VEGFR-2). In a phase 2 open label study on PTK787 in GISTs resistant to imatinib [44], 13% patients achieved PR, 8 (53%) had SD for 3 months or longer. The clinical benefit rate (PR + SD) was 67%. The dose of 1,250 mg daily was generally well tolerated.

## Novel agents

### mTOR inhibitors

Novel approaches to overcome resistance to TKIs in GIST include targeting multiple levels of the signal transduction cascade intracellularly by combining agents (Table 3). This has been done by combining a kinase inhibitor such as imatinib with an mTOR inhibitor everolimus. [57]. In this phase 1/2 trial Schoffski *et al.* reported stable disease in 36%, PR in 2% and stable disease (SD) in 43% patients who had progressed after imatinib and sunitinib/other tyrosine kinase inhibitor. All these patients were treated with imatinib 600 mg/day plus everolimus 2.5 mg/day. Another phase 1/2 trial showed SD in 8 out of 31 patients in the trial [58].

**Table 3:** Novel agents for GIST

Study Drug	Class of Drug	Disease	Dosage	Clinical Trial	No. of patients	Response	Reference	
Everolimus	mTOR inhibitor	TKI resistant	Everolimus 2.5 mg/day with Imatinib	Phase 1/2	58	PFS 29%	[57]
Everolimus	mTOR inhibitor	Refractory GIST	10 mg/day	Phase 1/2	15	ORR27%	[59]
Sirolimus	mTOR inhibitor	TKI resistant with PDGFRA-D842V.	Sirolimus (2–3 mg/day) with a TKI.	Retrospective	3	Signs of antitumor activity.	[60]
Ganetespib	Hsp90 inhibitor	Following failure of prior therapy	200 mg/m2 IV qweek for 3 wks of a 28 day cycle.	Phase 2	26	SD 52%	[61]
Retaspimycin (IPI 504)	Hsp90 inhibitor	Following failure of TKIs	400 mg/m2 weekly for 2 doses in 21‒day cycles	Phase 3	47	Too toxic	[62]
Perifosine	Akt pathway inhibitor	Imatinib-resistant	2 doses of perifosine - 100 mg daily or 900 mg qweekly with daily imatnib	Phase 2	41	Minimal activity	[63]

Abbreviations: *SD* stable disease; *PFS* progression free survival; *ORR* overall response rate.

Another mTOR inhibitor, sirolimus, as a single agent has also been reported [59,60]. Richter and co-workers showed response efficacy (complete response, partial response or stable disease) in 27% refractory and heavily pre-treated patients [59]. Piovesan and colleagues reported anti-tumor activity of sirolimus in combination with TKIs in 3 patients with PDGFRA-D842V metastatic GIST. Of these 3 patients, two were progressing on imatinib, while the third patient was treated with imatinib and sirolimus upfront [60].

### Hsp90 inhibitor

Other strategies that are being explored include the inhibition of other pathways involving KIT or PDGFRA oncoproteins, such as the heat shock protein-90 (Hsp90) chaperon system. By inhibiting Hsp90, preclinical and early clinical studies have already documented antineoplastic effects on resistant GIST both *in vitro* and in patients with progressive disease [64,65].

Ganetespib (STA 9090) is a potent, synthetic inhibitor of Hsp90. It has an improved safety profile relative to 1st-generation Hsp90 inhibitors and has promising signals of antitumor activity in early clinical studies, including one patient with PDGFRA D842V mutant GIST. Demetri and co-workers [61] enrolled patients with advanced GIST who failed prior therapy to receive ganetespib (200 mg/m2) as a 1 hour IV infusion weekly for 3 weeks of a 28 day cycle. Toxicities reported in more than 20% patients were grade 1–2 and included diarrhea, fatigue, nausea, vomiting, increased alkaline phosphatase, headache, insomnia, and abdominal pain. Fifty two percent (12/23) evaluable patients had stable disease. However, analysis of client proteins in paired tumor biopsies (4 patients) did not show prolonged inhibition of activated KIT or its downstream pathways. The data suggest that once-weekly treatment schedule is not optimal for inhibition of KIT.

Retaspimycin hydrochloride (IPI504, a Hsp90 inhibitor) is another Hsp90 inhibitor [62]. A clinical study was terminated early due to a higher mortality rate in the IPI504 arm compared to the placebo arm. IPI504 was not well tolerated in this patient population. There was a higher rate of Grade 3 and 4 adverse events, including LFT abnormalities, in the IPI504 arm.

### Perifosine

Other drugs that have been tried recently include perifosine [63]. The addition of perifosine to imatinib showed minimal activity in imatinib-refractory GIST.

## Conclusion and future directions

In summary, it is clear that the deeper scientific understanding of GIST has led to the development of novel therapeutic tools such as imatinib and sunitinib to disable the malignant GIST cells. With improved technology and rational molecular targeting, this translation of science into applied therapeutics should continue to move forward at a very rapid pace. It is foreseeable that more agents with novel mechanisms of action and targeting different pathways will be studied for GIST therapy.

## Competing interests

The authors have no conflicts of interests.

## Author’s contributions

SA, RG, BL contributed to data preparation. GL and DL were involved in concept design, data collection, and manuscript preparation. All authors reviewed and assisted in revising the manuscript. All authors read and approved the final manuscript
